# Physical Therapy Interventions: A Case Report of Building Strength, Confidence, and Mobility in a Seven-Year-Old With Congenital Femoral Deficiency With Coxa Vara

**DOI:** 10.7759/cureus.55662

**Published:** 2024-03-06

**Authors:** Ishwin Kaur B Bagga, Raghumahanti Raghuveer, Swarna Singh

**Affiliations:** 1 Department of Neuro-Physiotherapy, Ravi Nair Physiotherapy College, Datta Meghe Institute of Higher Education & Research, Wardha, IND

**Keywords:** case report, strengthening, play therapy, gait training, balance training, limb-length discrepancy, congenital coxa vara, congenital femoral deficiency

## Abstract

Congenital femoral deficiency (CFD) and congenital coxa vara (CCV) are rare conditions characterized by abnormal development of the femur and hip joint, respectively. This case report documents the rehabilitation journey of a seven-year-old child diagnosed with CFD and CCV, highlighting the efficacy of physical therapy interventions in enhancing strength, balance, normal gait patterns, confidence, and mobility. Through a comprehensive physiotherapy regimen tailored to the specific needs of the patient, significant improvements in muscle strength, joint stability, and functional mobility were observed over the course of treatment. Moreover, the implementation of targeted exercises and adaptive strategies not only facilitated physical gains but also contributed to bolstering the child’s confidence and overall quality of life. This case underscores the pivotal role of physiotherapy in addressing the complex challenges associated with congenital orthopedic anomalies, ultimately fostering independence and well-being in pediatric patients.

## Introduction

Femoral dysplasia is a condition characterized by a range of developmental abnormalities, including femoral deficits varying from slight shortening to complete absence, as well as structural changes such as coxa vara, pseudarthrosis, and hip dislocation [1]. Congenital femoral deficiency (CFD), also known as proximal femoral focal deficiency, is an uncommon birth defect affecting the hip, femur, and knee. The degree of deformity can vary, ranging from modest shortening to proximal or distal deficiency, or in extreme cases, total loss of the femur [2,3]. CFD has an incidence of 1.1-2.0 per 100,000 live births [1]. Longitudinal reduction syndromes are characterized by distinct deformity patterns, with the most evident anomaly taking the front stage. Gillespie’s classification system takes into account longitudinal deficiency, proximal femur morphology, and hip stability [4-6]. Choosing the best therapy for a patient depends on criteria such as surgical competence, prosthetic availability, and patient and family preferences. The treatment options include surgical lengthening or prosthetic management. The surgical method can be done by using an Ilizarov ring fixator, or even in a few cases, after a failed attempt at prosthetic management, Syme’s amputation is done [4,7,8].

Congenital coxa vara (CCV) is a developmental anomaly that causes a reduced femoral neck-shaft angle, a shortened femoral neck, and a short lower limb (LL). CCV is thought to be caused by a deficiency in enchondral ossification of the femoral neck’s medial half. The specific etiology is unclear [9,10]. CCV is a very uncommon malformation, occurring in around one in every 25,000 live births. Patients with CCV frequently exhibit gait problems or limb length asymmetry [9]. Coxa vara can be effectively treated surgically by adjusting the Hilgenreiner epiphyseal angle to around 35 degrees or the neck shaft angle to more than 120 degrees to prevent the recurrence of the deformity [11,12]. Various techniques have been utilized to fix the femoral osteotomy, including tension band wire, angle blade plates, dynamic sliding hip screws, and external fixators [11,13].

Nonoperative management of CFD mainly includes physiotherapy, bracing, and the use of prosthetics. Physical therapy includes the use of thermal therapy, massage, range of motion (ROM) exercises, strengthening exercises, and gait training [14,15]. Physiotherapy for coxa vara deformity is prescribed as a nonoperative as well as a postoperative surgical procedure [16]. Physical therapy includes bracing, positioning, and ROM exercises. Weight-bearing and gait training are the key components of the intervention [9,17].

## Case presentation

Patient information 

A seven-year-old child was brought to the neuro OPD by his parents in December 2023. The patient’s history was narrated by his mother. The mother noticed a delay in the milestones since the age of two and a half years, with the child having difficulty walking independently and being able to only crawl. The patient exhibited a persistent pelvic tilt toward the left side, with his left leg shorter and the left knee positioned higher than the right. Initial evaluation by a local practitioner in Yavatmal Hospital recommended surgery for left femur head correction, which was deferred due to financial constraints. Parents facilitated the child’s standing and walking with support at home. However, by the age of six, the child continued to face mobility challenges and was unable to participate in physical activities with peers. A subsequent visit to Yavatmal Hospital resulted in a surgical procedure of soft tissue reconstruction and Achilles tendon lengthening of the left ankle, yet the discrepancy in leg lengths persisted post-surgery. There was no documentation of the treatment with the parents. Parents then visited the Acharya Vinoba Bhave Rural Hospital with the same complaints. X-ray findings identified that the patient had a CFD. The patient was referred for physiotherapy for management and was suggested to utilize the heightened shoes for the left LL.

Clinical findings

Informed consent was obtained by the child’s mother before the examination. Cranial nerve examination, reflexes, motor tone assessment, and sensory examination were intact. Romberg’s test, single-leg stance test, functional reach test, and tandem stance test were positive. Upper limb ROM was normal. There was a significant decrease in the LL ROM bilaterally. Manual muscle testing (MMT) assessment showed reduced strength in bilateral LL. Table 1 shows true limb length and segmental limb length measurements. Table 2 shows the pre- and post-rehabilitation findings of the ROM assessment. Table 3 shows the MMT assessment of pre- and post-rehabilitation.

**Table 1:** Limb length assessment

Limb length	Right	Left
True	52 cm	47 cm
Segmental: From the greater trochanter of the femur to the patella	27 cm	22 cm
From the patella to the lateral malleolus	25 cm	25 cm

**Table 2:** ROM assessment of bilateral LL LL, lower limb; ROM, range of motion

Joint	Right				Left			
	Pre-rehabilitation		Post-rehabilitation		Pre-rehabilitation		Post-rehabilitation	
	Active	Passive	Active	Passive	Active	Passive	Active	Passive
Hip: Flexion	0-80°	0-85°	0-104°	0-116°	0-78°	0-85°	0-100°	0-112°
Extension	0-6°	0-10°	0-18°	0-25°	0-5°	0-10°	0-15°	0-22°
Abduction	0-24°	0-30°	0-40°	0-45°	0-20°	0-27°	0-35°	0-42°
Adduction	0-20°	0-25°	0-30°	0-30°	0-20°	0-25°	0-30°	0-30°
Knee: Flexion	0-82°	0-95°	0-110°	0-132°	0-80°	0-90°	0-108°	0-130°
Extension	82°-0	95°-0	110°-0	132°-0	80°-0	90°-0	108°-0	130°-0
Ankle: Plantarflexion	0-32°	0-40°	0-45°	0-50°	0-30°	0-38°	0-45°	0-50°
Dorsiflexion	0-10°	0-15°	0-15°	0-20°	0-8°	0-15°	0-15°	0-20°

**Table 3:** MMT assessment of bilateral LL Oxford Scale: Grade 0: no contraction; Grade 1: flicker of movement; Grade 2: full ROM with gravity; Grade 3: full ROM against gravity; Grade 4: full ROM against gravity with minimum resistance; Grade 5: full ROM against gravity with maximum resistance LL, lower limb; MMT, manual muscle testing; ROM, range of motion

Muscle	MMT grade (right)		MMT grade (left)	
	Pre-rehabilitation	Post-rehabilitation	Pre-rehabilitation	Post-rehabilitation
Hip: Flexors	2	5	2	5
Extensors	2	5	2	5
Abductors	3	5	3	5
Adductors	3	5	3	5
Knee: Flexors	2	5	2	5
Extensors	2	5	2	5
Ankle: Plantarflexors	3	5	3	5
Dorsiflexors	3	5	3	5

Diagnostic assessment

**Figure 1:**
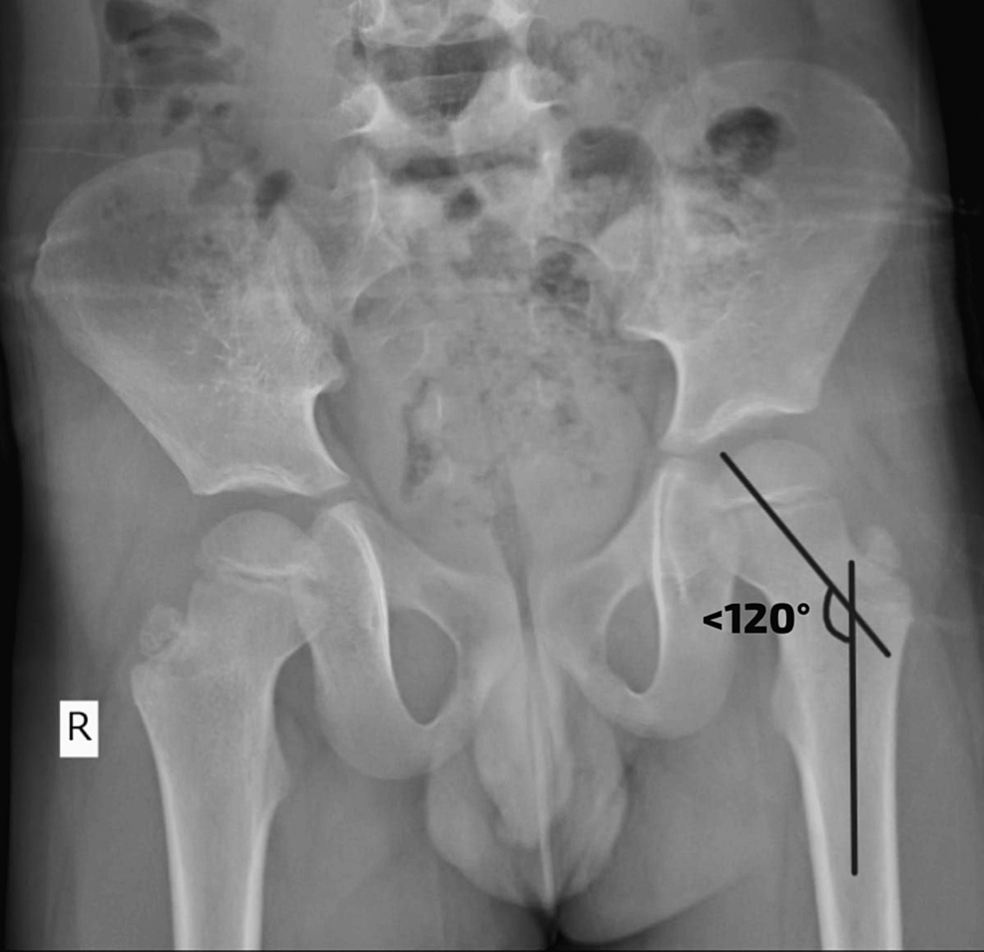
CCV deformity CCV, congenital coxa vara

**Figure 2:**
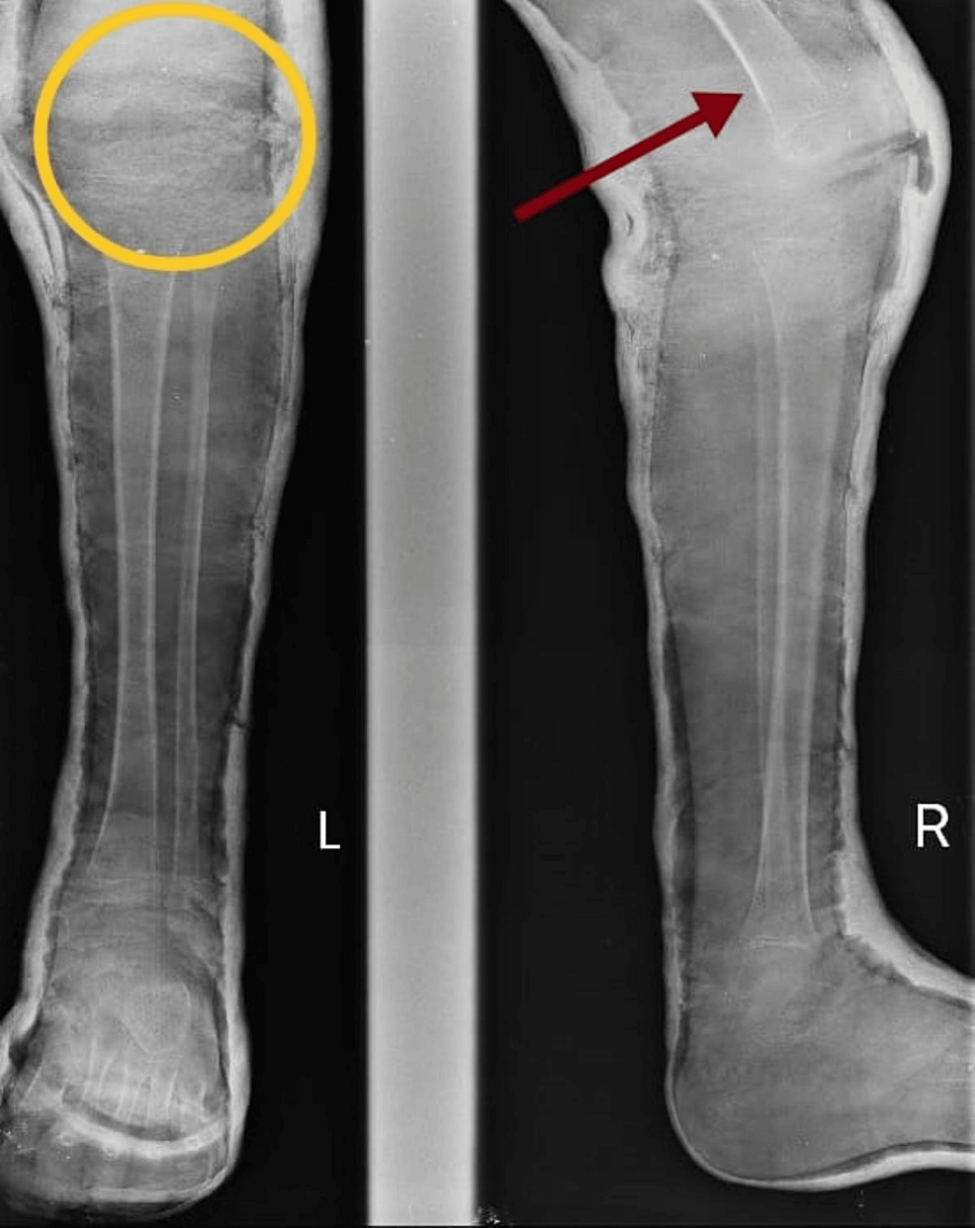
Reduced length of the left femur (CFD) The yellow circle shows the upper placement of the knee joint in the left leg. The red arrow shows the right femur, which is longer than the left femur. CFD, congenital femoral deficiency

The patient had CFD and coxa vara. The investigation X-ray was done to diagnose the conditions. Figure 1 shows a CCV deformity. Figure 2 shows the reduced length of the left femur bone.

Physiotherapy intervention 

The patient reported complaints of impaired balance, an abnormal gait pattern, pelvic instability, and reduced strength. Consequently, a physiotherapy protocol was planned. Table 4 shows the planned physical therapy protocol for the patient.

**Table 4:** Planned physical therapy protocol for the patient LL, lower limb; ROM, range of motion

Problem list	Goals	Intervention	Dosage	Rationale
Diagnostic awareness	Educate parents about their child’s condition and the importance of exercises	Explain treatment goals and demonstrate exercises	At the beginning of therapy and as per need	Empowers both child and parent to participate actively in therapy and home exercises
Psychosocial support	Enhance emotional well-being	Encourage the expression of feelings and provide positive reinforcement	Throughout the therapy session	Supports emotional resilience and coping skills for the child
Muscle weakness	Increase LL strength	Resistance training: body weight shift exercises and theraband exercises	Three times per day for 15-20 minutes	Strengthening muscles will support functional activities and prevent compensatory movements
Balance impairment	Improve balance and proprioception	Balance exercises: single-leg stance, parallel bars, Swiss ball exercises, and obstacle walking	Twice a day for 10-15 minutes	Enhances stability, reduces fall risk, and improves functional abilities
Abnormal gait pattern	Normalize gait mechanics	Gait training: walking drills, step-ups, stairs climbing, and treadmill walking with an assistive device	Two times per day for 10 minutes	Correcting gait mechanics promotes efficient and symmetrical movement patterns and promotes independence in activities of daily living
Pelvic instability	Enhance pelvic stability	Pelvic stabilization exercises: pelvic tilts on a Swiss ball and treadmill	Twice a day for 10 minutes	Improves alignment and control of the pelvis, supporting proper gait mechanics
Tightness of the LL muscles	Prevent stiffness and improve movement	Stretching: hamstring stretch, hip adductors stretch, and Achilles tendon stretch	Two times per day, with 30-second holds during each stretch	Improves ROM and prevents stiffness
Functional inability	Improve functional movement patterns	Functional exercises: step-ups, lunges, and functional reach-outs on the Swiss ball	Thrice a day, incorporate into daily activities	Enhances ability to perform daily tasks and participate in recreational activities
Disinterest in play activities	Promote engagement and enjoyment	Play therapy: ball catching and throwing, kicking exercises, and object pick-up activities	Incorporate into every session	Increases motivation, adherence, and enjoyment of therapy sessions
		Relaxation, including deep breathing		Promotes relaxation, reduces muscle tension, and supports overall well-being
Home exercise program	Reinforce therapy gains at home	Continue the exercises taught by the therapist	Two times daily for 30 minutes	Enhances continuity of care and reinforces progress made during therapy sessions

Outcome measures

**Figure 3:**
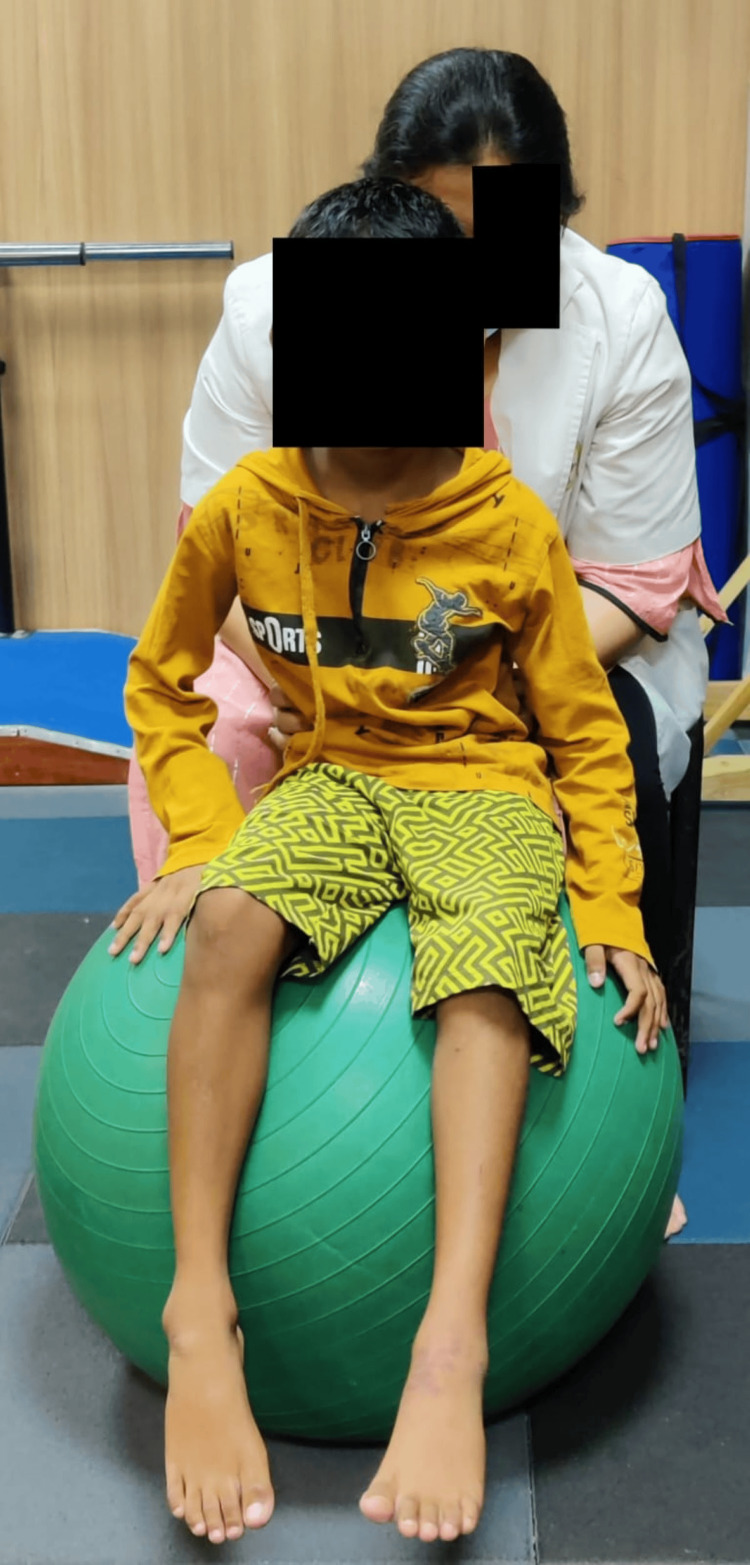
Pelvic tilts on a Swiss ball

**Figure 4:**
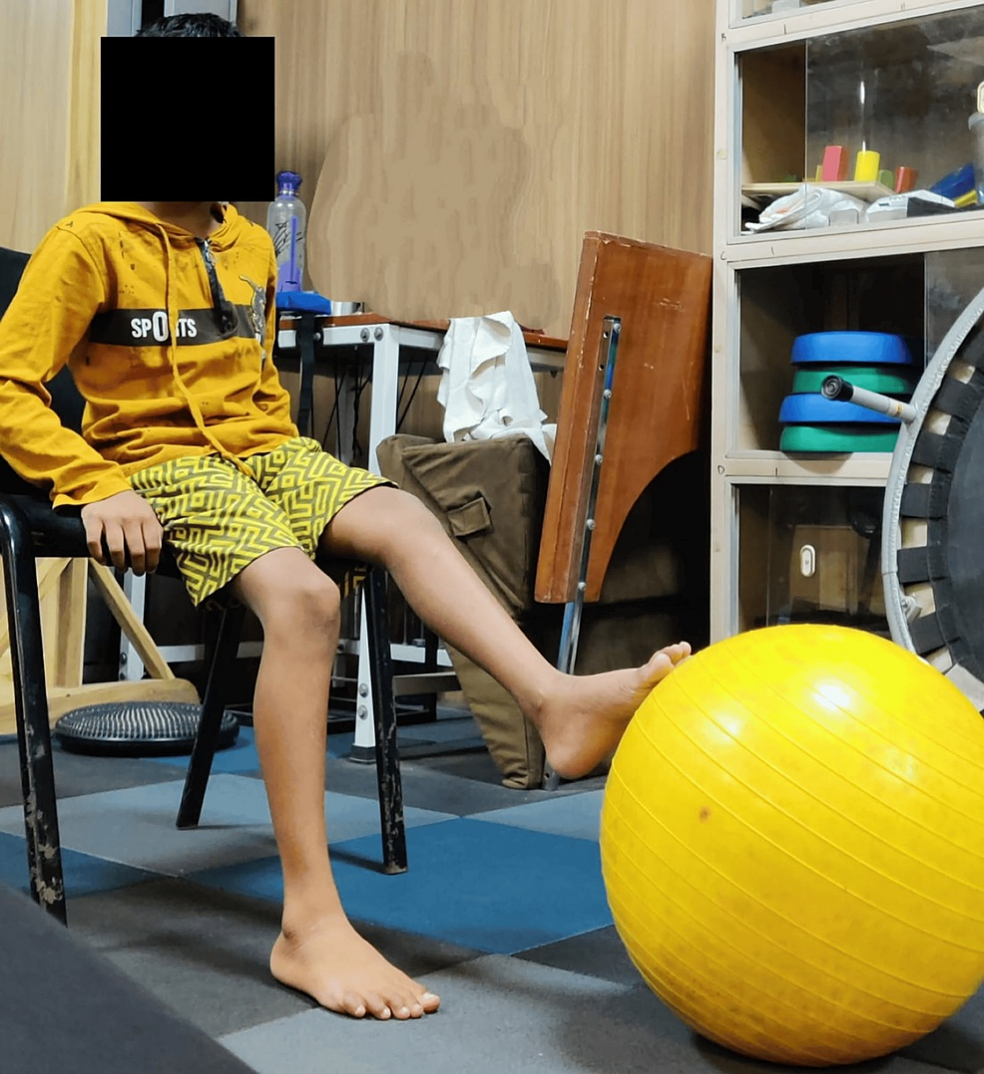
Play therapy: ball kicking exercises

**Figure 5:**
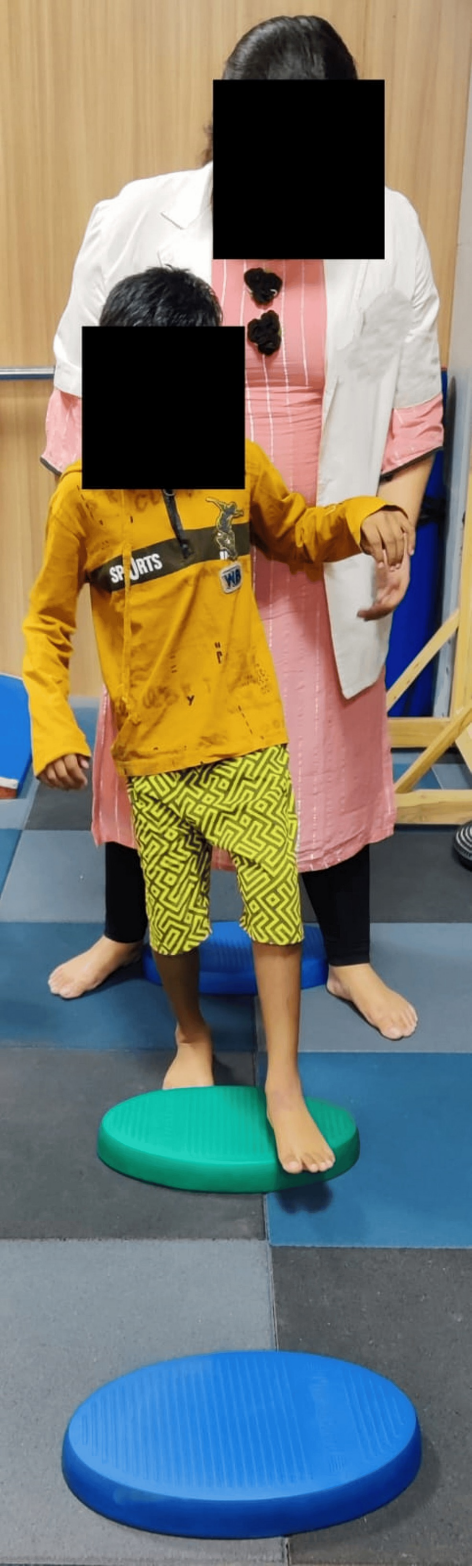
Gait training: walking over obstacles

**Figure 6:**
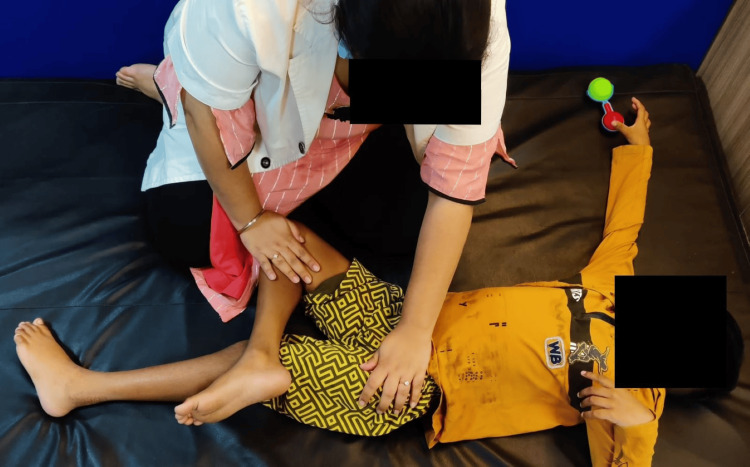
Stretching for hip adductors

A functional gait assessment was done to assess the gait of the patient. The Berg Balance Scale and functional reach test were used for the assessment of balance. SF-36 was used to assess the quality of life. Outcomes were assessed on day one before starting the rehabilitation and after four weeks post-rehabilitation. Table 5 shows the outcomes used for the patient to assess progression. Figure 3 shows pelvic tilts given to the patient on a Swiss ball. Figure 4 shows play therapy (ball kicking). Figure 5 shows gait training (walking over obstacles). Figure 6 shows stretching given for hip adductors.

**Table 5:** Outcome measures used for patient

Outcome measure	Pre-intervention	Post-intervention
Functional gait assessment	14/30 (fall risk is high)	24/30 (ambulatory with low risk of fall)
Berg Balance Scale	10/56 (high fall risk)	42/56 (low fall risk)
Functional reach test	6.7 inches (limited functional balance)	11.1 (adequate functional balance)
SF-36	36/100 (moderately affected quality of life)	78/100 (better quality of life)

## Discussion

CFD encompasses various forms of longitudinal deficiency, such as proximal focal femoral deficiency, lateral distal femoral hypoplasia, knee cruciate ligament deficiency, and involvement of the contralateral limb [18,19]. Treatment aims to optimize function through limb equalization and correction of deformities, utilizing strategies ranging from nonsurgical approaches to lengthening, shortening, and complex limb reconstruction [19]. Coxa vara is a very rare condition caused by a faulty femoral epiphyseal plate. The femoral head and acetabulum appear to be normal at birth and throughout the first few months of life. However, when bone development and weight bearing occur, the mechanics of the hip joint change significantly, resulting in secondary alterations and joint incongruity [20]. In 2013, Monsell concluded in his study that physical therapy plays a vital role in CFD patients, both in cases where surgical procedures are not required and in cases that require surgical procedures. It helps in improving strength, balance, gait patterns, and overall well-being [4]. In this case, the patient had reduced strength, balance impairments, gait abnormalities, and pelvic instability. The physiotherapy protocol was focused on strength training and improving balance and gait patterns. Psychosocial support and counseling for both child and parents helped significantly improve the prognosis and results. Patients’ mental and physical health improved significantly.

## Conclusions

The case report highlights the significant role of physical therapy interventions in enhancing strength, confidence, and mobility in a seven-year-old with CFD and CCV. Through tailored exercises and interventions, the patient demonstrated notable improvements in muscle strength, functional abilities, balance and gait impairment, and overall well-being. This underscores the importance of early and targeted interventions for pediatric patients with complex orthopedic conditions, emphasizing the potential for physical therapy to empower individuals and optimize their quality of life.

## References

[1] Lloyd-Roberts GC, Ratliff AH, Graham Apley A (2014). Hip Disorders in Children: Postgraduate Orthopaedics Series. Am J Roentgenol.

[2] Paley D, Chong DY, Prince DE (2016). Congenital femoral deficiency reconstruction and lengthening surgery. Pediatric Lower Limb Deformities.

[3] (2024). PFFD - definition, classification and management. http://myplace.frontier.com/.

[4] Monsell FP, Bintcliffe FA, Evans C, Hughes R (2013). Management of congenital femoral deficiency. Early Hum Dev.

[5] Gillespie R, Torode IP (1983). Classification and management of congenital abnormalities of the femur. J Bone Joint Surg Br.

[6] Boden SD, Fallon MD, Davidson R, Mennuti MT, Kaplan FS (1989). Proximal femoral focal deficiency. Evidence for a defect in proliferation and maturation of chondrocytes. J Bone Joint Surg Am.

[7] Ilizarov GA (1989). The tension-stress effect on the genesis and growth of tissues: Part II. The influence of the rate and frequency of distraction. Clin Orthop Relat Res.

[8] Ilizarov GA (1989). The tension-stress effect on the genesis and growth of tissues. Part I. The influence of stability of fixation and soft-tissue preservation. Clin Orthop Relat Res.

[9] Hart ES, Grottkau BE, Marino JC (2007). Congenital coxa vara deformity. Orthop Nurs.

[10] Oh CW, Thacker MM, Mackenzie WG, Riddle EC (2006). Coxa vara: a novel measurement technique in skeletal dysplasias. Clin Orthop Relat Res.

[11] Srisaarn T, Salang K, Klawson B, Vipulakorn K, Chalayon O, Eamsobhana P (2019). Surgical correction of coxa vara: evaluation of neck shaft angle, Hilgenreiner-epiphyseal angle for indication of recurrence. J Clin Orthop Trauma.

[12] Pauwels F (2024). Biomechanics of the Normal and Diseased Hip: Theoretical Foundation, Technique and Results of Treatment An Atlas. https://link.springer.com/book/10.1007/978-3-642-66212-6..

[13] Amstutz HC, Freiberger RH (1962). Coxa vara in children. Clin Orthop.

[14] Piechocka E, Wrzesiński B, Wojtczak P, Ziółkowska A, Ciecierska D (2018). Physiothreapeutic treatments in infants with congenital hip dysplasia. J Educ Health Sport.

[15] Cooper A, Fernandes JA (2017). Evidence-based treatment for congenital femoral deficiency. Paediatric Orthopaedics.

[16] Taşar M, Eyileten Z, Kasımzade F, Uçar T, Kendirli T, Uysalel A (2014). Camptodactyly-arthropathy-coxa vara-pericarditis (CACP) syndrome. Turk J Pediatr.

[17] Ippolito E, Farsetti P, Benedetti Valentini M, Fichera A (2016). Two-stage surgical treatment of complex femoral deformities with severe coxa vara in polyostotic fibrous dysplasia. JBJS Essent Surg Tech.

[18] Pappas AM (1983). Congenital abnormalities of the femur and related lower extremity malformations: classification and treatment. J Pediatr Orthop.

[19] Nossov SB, Hollin IL, Phillips J, Franklin CC (2022). Proximal femoral focal deficiency/congenital femoral deficiency: evaluation and management. J Am Acad Orthop Surg.

[20] Calhoun JD, Pierret G (1972). Infantile coxa vara. Am J Roentgenol Radium Ther Nucl Med.

